# Hypertensive Heart Disease in the Nigerian Population: Prevalence, Phenotypes, and Cardiovascular Comorbidities

**DOI:** 10.7759/cureus.82060

**Published:** 2025-04-11

**Authors:** Olugbenga O Abiodun, Tina Anya, Ibrahim Salau, Olalekan Ogunsakin, Victor Adekanmbi

**Affiliations:** 1 Internal Medicine/Cardiology, Federal Medical Centre, Abuja, NGA; 2 Basic Biomedical Sciences (Pathology), Touro University College of Osteopathic Medicine (TouroCOM), New York, USA; 3 Obstetrics and Gynecology, University of Texas Medical Branch at Galveston, Texas, USA

**Keywords:** hypertensive heart disease, left ventricular diastolic dysfunction, left ventricular hypertrophy, nigerians, prevalence

## Abstract

Objective

There is a lack of studies on the phenotyping of hypertensive heart disease (HHD) that examine both left ventricular (LV) structure and function in patients with essential hypertension (HTN) in sub-Saharan Africa. In this study, we investigated the prevalence of HHD, its phenotypic characteristics, and associated cardiovascular (CV) comorbidities by analyzing both LV structure and function. This can significantly enhance the understanding of HHD in the Nigerian population.

Methods

This cross-sectional study involved 1,799 patients diagnosed with essential HTN, who were recruited from the Federal Medical Centre Abuja Hypertension Registry between 2016 and 2021. HHD was defined as the presence of abnormal LV geometry and/or LV diastolic dysfunction (LVDD), as assessed by echocardiography in accordance with the guidelines set by the American Society of Echocardiography and the European Association of CV Imaging. Abnormal LV geometry was characterized by either concentric remodeling, which is defined as a normal left ventricular mass index (LVMI) with a relative wall thickness (RWT) > 0.42, or left ventricular hypertrophy (LVH), which is indicated by an increased LVMI (> 95 g/m² in women and > 115 g/m² in men) with an RWT > 0.42. Patients with secondary HTN and those with HTN during pregnancy were excluded from the study.

Results

The mean age of the study participants was 55.3±13.0 years and the majority were female patients (55.9%). The prevalence of HHD, concentric LV remodeling, LVH, and LVDD was 90.8%, 38.2%, 47.4%, and 61.6%, respectively. After multivariate analysis, heart failure (adjusted odds ratio (OR): 9.71, confidence interval (CI) 6.20-15.23, p<0.001), stroke (OR: 1.59, CI 1.01-2.52, p=0.045), LVDD (OR: 2.01, CI 1.61-2.50, p<0.001), and female sex (OR: 1.47, CI 1.20-1.80, p<0.001) were independently and positively associated with LVH. Similarly, LVH (OR: 3.51, CI 2.53-4.87, p<0.001), diabetes mellitus (OR: 1.83, CI 1.37-2.44, p<0.001), concentric LV remodeling (OR: 2.18, CI 1.58-3.01, p<0.001), stroke (OR: 1.87, CI 1.06-3.32, p=0.032), and age ≥60 years (OR: 3.92, CI 3.09-4.96, p<0.001) were independently and positively associated with LVDD.

Conclusion

Our study showed a high prevalence of HHD with LVH and LVDD as common phenotypes in our hypertensive cohort. These findings suggest that the widespread use of echocardiography should be promoted to aid the early diagnosis of HHD.

## Introduction

Hypertensive heart disease (HHD) arises from the changes in cardiac structure and function and may include abnormal geometry, fibrosis, diastolic dysfunction, and vascular alterations in individuals with longstanding hypertension (HTN) [1]. HTN is the number one risk factor for mortality globally, with an estimated 1.39 billion cases in 2010, and this is expected to substantially exceed 1.6 billion by 2025 [2-4]. In western sub-Saharan Africa (SSA), HHD had the highest age-standardized disability adjusted life years (DALYs) in 2022 at 593.8 per 100,000 after ischemic heart disease and all stroke subtypes while high systolic blood pressure (SBP) had the largest number of attributable age-standardized cardiovascular (CV) DALYs at 3,330.4 per 100,000 [3].

An increase in peripheral vascular resistance leads to abnormal geometric adaptation of the heart in the form of remodeling or hypertrophy, with the eventual development of heart failure (HF) [5,6]. Life-threatening arrhythmias and sudden death may result from interstitial fibrosis, the main histological feature of HHD diagnosed by advanced imaging techniques such as cardiac magnetic resonance (CMR) [7]. The mechanisms of arrhythmogenesis may involve inhomogeneity and conduction delay, which may lead to conduction abnormalities and reentrant arrhythmias [1,5,6]. Coronary macro- or microvascular changes involving medial hypertrophy and perivascular fibrosis also put patients with HHD at an increased risk of HF, coronary heart disease (CHD), and death [5,6].

Left ventricular hypertrophy (LVH) and left ventricular diastolic dysfunction (LVDD) are the most common abnormalities in HHD, accounting for >40% and 40-85%, respectively, in patients with HTN [1,8]. They are of great significance because independently, both are predictors of adverse CV outcomes in hypertensives [1]. Other structural and functional abnormalities seen in HHD are less common [1]. In SSA, literature on the combination of LV geometric patterns and LVDD in HTN is limited to the best of our knowledge. Most SSA studies on HTN have focused on LV geometric patterns only, possibly because of the ease of evaluation of geometric patterns and the contentious reliability of algorithms of diastolic function compared with invasive measurements [1,9-12]. In an echocardiographic study of newly diagnosed hypertensive patients in Nigeria, Aje et al. [10] focused solely on LV geometric patterns. On the other hand, Adamu et al. [12] investigated LVDD only using pulsed wave Doppler echocardiography. Furthermore, in a study conducted by Nkoke et al., the predominance of HHD among patients with cardiac conditions in Southwest Cameroon was examined. However, the criteria used to define LV geometric patterns and diastolic function were narrow and unclear [13]. Assessing HHD with both LV geometry and LVDD confers the advantage of early recognition of early stages of HHD characterized by delayed relaxation and concentric remodeling [1]. By evaluating HHD using both LV geometry and LVDD, our study captures the true burden throughout the course of HHD from early to late stages. Moreover, studies that have been used to assess the burden of HHD in SSA have been population-based using SBP as a surrogate [2,14]. SBP alone is insufficient for assessing HHD given that other phenotypes of HTN such as diastolic and masked HTN also contribute to hypertensive-mediated cardiac damage [3,4,8]. The use of SBP in these studies may be because the basic tools to diagnose HHD such as electrocardiography (ECG) and echocardiography are relatively expensive and not readily available for population-based screening. Data on the burden of HHD derived from these studies can therefore not be representative given the additional burden of HF, stroke, CHD, and all-cause mortality that HHD poses [1].

This study aims to provide important data on the prevalence, phenotypes, and comorbidities of HHD in the Nigerian population based on the presence of LV geometric patterns and LVDD. This will provide data to bridge the gap caused by the use of SBP as a surrogate for HHD. Furthermore, this information will assist policymakers in recognizing the true burden of HHD and may promote the development of population-wide initiatives and policies. Policies such as implementing early and subsidized echocardiographic testing, especially in community settings, could help reduce the morbidity and mortality associated with HHD.

## Materials and methods

Methods and subjects

**Figure 1:**
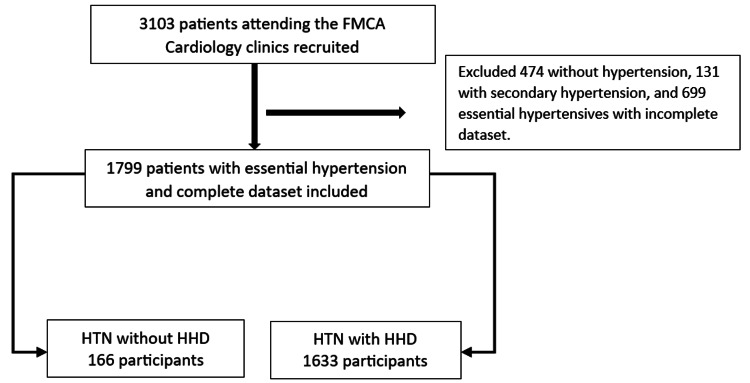
Data flow diagram of exclusion criteria to determine the study population included in the study analysis FMCA: Federal Medical Centre Abuja; HTN: Hypertension; HHD: Hypertensive heart disease.

Consenting patients from the Federal Medical Centre Abuja Hypertension Registry (FMCAHR) were included in this cross-sectional study. The FMCAHR recruited all consenting patients (n= 3,103) who attended the Cardiology clinics of the Federal Medical Centre Abuja (FMCA) between 2016 and 2021. FMCA is a tertiary healthcare institution that caters to the residents of Abuja and beyond. Data was collected using detailed history taking, physical examination, anthropometry (weight, height, waist circumference, and body mass index), first-degree family history of HTN and diabetes mellitus (DM), comorbidities (dyslipidemia, DM, obesity, atrial fibrillation (AF), cerebrovascular disease or stroke, coronary artery disease (CAD), chronic kidney disease (CKD), and HF), routine blood and urine tests, chest radiography, ECG, and echocardiography. Additional tests such as abdominopelvic ultrasound, thyroid function test, urinary metanephrines, and serum cortisol were carried out in suspected cases of secondary HTN. Blood pressure was measured by physicians using a mercury sphygmomanometer (Accosson, London, UK). One thousand seven hundred and ninety-nine (1,799) essential hypertensive patients aged 18 years and above were analyzed (Figure 1). Patients without HTN, those with secondary HTN, and those with missing data were excluded from this analysis. All patients gave informed consent, and the approval of the hospital’s ethics research committee was obtained (FMCABJ/HREC/2017/009).

Echocardiography

Echocardiography was performed on all patients by the first two authors who are experienced cardiologists at patients’ first visit, and subsequently as appropriate using General Electric Model Vivid E9, with a M5S probe (GE Healthcare, Horten, Norway) or General Electric, Model S6, with a M4S probe (GE Healthcare, Horten, Norway). Measurements were taken following recommendations of the American Society of Echocardiography (ASE) and the European Association of CV Imaging (EACVI) [15]. Echocardiography was used to determine increased left ventricular mass index (LVMI) as >95 g/m^2^ in women and >115 g/m^2^ in men according to the American Society of Echocardiography [15]. Left ventricular internal diameter in diastole (LVIDD), left ventricular posterior wall thickness at end-diastole (PWTD), and interventricular septal thickness at end-diastole (IVSTD) were measured to calculate LVMI. LVMI was calculated using the linear cube formula of the American College of Cardiology which aligns with necropsy measurements [15]: \[ \text{LVMI} = \frac{0.8 \times \left(1.04 \times \left[(\text{LVIDD} + \text{PWTD} + \text{IVSTD})^3 - \text{LVIDD}^3\right]\right) + 0.6}{\text{BSA}} \]

The relative wall thickness (RWT) was calculated with the formula [15]: \[ \text{RWT} = \frac{2 \times \text{PWTD}}{\text{LVIDD}} \]. Mitral peak E wave velocity (E), mitral peak A velocity (A), E/A ratio, and tissue doppler-derived mitral annular e’ velocity (e’) and E/e’ were measured to grade LVDD. LVDD was defined as the presence of any of the following grades of LVDD: grade 1- lateral E' < 0.1 m/s and E/A < 0.8; grade 2- E/A of 0.8-< 2 and E/e' of 10-14, and grade 3- E/A > 2 and E/e' > 14 [1,16].

Definitions

HHD is defined as the presence of abnormal LV geometry, and or LVDD by echocardiography [1,15,16]. Abnormal LV geometry is defined by the presence of concentric LV remodeling or left ventricular hypertrophy [1,15]. Concentric LVH is defined as increased LVMI with RWT of >0.42, while eccentric LVH is defined as increased LVMI with RWT of ≤0.42 [15]. Concentric LV remodeling is defined as normal LVMI with RWT >0.42, while normal geometry is defined as normal LVMI and RWT of ≤0.42 [15]. Essential HTN was diagnosed when office BP was persistently elevated at ≥140/90 mmHg, as determined by a physician, or if the patient was on HTN medications. DM was diagnosed by physicians, or if the patient was on DM medications. Dyslipidemia was diagnosed by physicians as abnormalities of total cholesterol, high-density lipoprotein, low-density lipoprotein and triglycerides. Obesity was diagnosed as body mass index of >30kg/m^2^. Heart failure with reduced ejection fraction (HFrEF), heart failure with mildly reduced ejection fraction (HFmrEF), heart failure with preserved ejection fraction (HFpEF), CKD, AF, CAD, and stroke were diagnosed by physicians according to guidelines of the American College of Cardiology, Chronic Kidney Disease Epidemiology Collaboration, European Society of Cardiology, and American Heart Association/American Stroke Association respectively [17-21]. Alcohol intake was defined as current intake of alcohol while tobacco use was defined as current and past active or passive use of tobacco or tobacco products.

Data analysis

Patients with missing data (n=699) were excluded using complete case analysis as well as patients without HTN (n=474) and those with secondary HTN (n=131). Categorical variables are expressed as numbers and percentages, while continuous variables are expressed as means ± standard deviation or as a range. The association of predictor variables of interest with the presence and absence of HHD was tested by chi-square for categorical variables and independent T-test for continuous variables. Fisher’s exact test was used for categorical variables with an expected cell size of less than 5. Multivariable logistic regression with model-fitting statistics was used to draw the association between statistically significant predictor variables from univariable models and the outcomes of interest. p-value <0.05 was taken as statistically significant as it indicates evidence against the null hypothesis. Data was analyzed using IBM SPSS Statistics for Windows, Version 26 (Released 2019; IBM Corp., Armonk, New York, United States).

## Results

Table 1 shows the baseline characteristics of all participants. The mean age of the study participants was 55.3±13.0 years with a preponderance of 1006 (55.9%) female patients. Patients with HTN only were younger compared to HHD patients (46.1±12.3 versus 56.2±12.8 years, p<0.001). The duration of HTN in months was longer in those with HHD compared with those with HTN only. DM was higher among HHD patients compared to those with HTN only. Eight hundred and fifty-three (47.4%) patients with HHD had LV hypertrophy followed by 688 (38.2%) for LV concentric remodeling, 572 (31.8%) for concentric hypertrophy, 281 (15.6%) for eccentric hypertrophy, and 258 (14.3%) for normal geometry (p<0.001). Patients with HHD had higher SBP, RWT, LVMI, mitral E/E’, and LVDD (p<0.05), while ejection fraction, mitral E/A, mitral E’, and eGFR were lower in patients with HHD (p<0.001). Patients with HHD had higher rates of HF, stroke, AF, CKD, and statin use (p<0.05). There was no significant difference between the two groups for DBP and CAD (p>0.05). Calcium channel blockers were the most common antihypertensive medication, followed by diuretics, angiotensin-converting enzyme inhibitors, and angiotensin receptor blockers (ARBs). In summary, Table 1 shows significant associations for age, duration of HTN, DM, dyslipidemia, SBP, LV geometric patterns, RWT, LVMI, LVH, EF, LVDD and parameters, eGFR, AF, HF, stroke, CKD, diuretics, ARBs, and statin use.

**Table 1:** Baseline characteristics of all participants ACEIs: Angiotensin-converting enzyme inhibitors; ARBs: Angiotensin receptor blockers; BMI: Body mass index; DBP: Diastolic blood pressure; E’: Tissue doppler-derived mitral annular e’ velocity; E/A ratio: Ratio of mitral peak E-wave velocity to mitral peak A-wave velocity; E/e’ ratio: Ratio of mitral peak E-wave velocity to tissue doppler-derived mitral annular e’ velocity; ECHO: Echocardiographic; HFmrEF: Heart failure with mildly reduced ejection fraction; HFpEF: Heart failure with preserved ejection fraction; HFrEF: Heart failure with reduced ejection fraction; HTN: Hypertension; HHD: Hypertensive heart disease; LVH: Left ventricular hypertrophy; SBP: Systolic blood pressure. *Fisher’s exact test, **Pearson chi-square, ***Independent t-test.

Variables	Total (n=1799)	HHD absent, n=166 (9.2%)	HHD present, n=1633 (90.8%)	p-value	Test value
Age (years)	55.3±13.0	46.1±12.3	56.2±12.8	<0.001	0.333***
Sex				0.199	1.649**
Male	793 (44.1%)	81 (48.8%)	712 (43.6%)		
Female	1006 (55.9%)	85 (51.2%)	921 (56.4%)		
Duration of hypertension (months)	88.7±99.6	56.6±75.2	92.0±101.2	<0.001	22.419***
Family history of hypertension	1039 (57.8%)	105 (63.3%)	934 (57.2%)	0.152	3.766**
Alcohol intake	570 (31.7%)	59 (35.5%)	511 (31.3%)	0.403	1.817**
Tobacco intake	108 (6.0%)	14 (8.4%)	94 (5.8%)	0.202	3.200**
Obesity (BMI≥30kg/m^2^)	888 (49.5%)	76 (45.8%)	812 (49.9%)	0.315	1.010**
Diabetes mellitus	363 (20.2%)	14 (8.4%)	349 (21.4%)	<0.001	15.782**
Dyslipidemia	1162 (70.0%)	99 (62.7%)	1063 (70.8%)	0.033	4.617**
First visit SBP (mmHg)	147.0±22.6	143.5±19.4	147.3± 22.9	0.036	4.102***
First visit DBP (mmHg)	89.0±14.1	89.0±11.8	89.0±14.3	0.966	5.959***
Blood pressure control	1255 (89.5%)	121 (91.0%)	1134 (89.3%)	0.819	0.400**
Concentric remodeling	688 (38.2%)	0 (0.0%)	688 (42.1%)	<0.001	113.247**
Concentric hypertrophy	572 (31.8%)	0 (0.0%)	572 (35.0%)	<0.001	85.252**
Eccentric hypertrophy	281 (15.6%)	0 (0.0%)	281 (17.2%)	<0.001	33.852**
Normal geometry	258 (14.3%)	166 (100%)	92 (5.6%)	<0.001	854.000**
Relative wall thickness	0.50±0.13	0.38±0.03	0.51±0.13	<0.001	117.130***
Left ventricular mass index (kg/m^2^)	109.6±37.3	85.3±13.9	112.1±38.0	<0.001	78.763***
ECHO Left ventricular hypertrophy	853 (47.4%)	0 (0%)	853 (52.2%)	<0.001	164.896**
Ejection fraction (%)	64.9±13.7	68.4±7.7	64.5±14.1	<0.001	28.678***
Mitral E/A ratio	1.07±0.74	1.24±0.33	1.06±0.77	<0.001	11.218***
Mitral E/e’	8.0±4.0	6.0±1.5	8.2±4.1	<0.001	42.811***
Mitral e’ (m/s)	0.11±0.05	0.14±0.03	0.10±0.05	<0.001	2.967***
LV diastolic dysfunction	1108 (61.6%)	0 (0.0%)	1108 (67.9%)	<0.001	293.924
Estimated glomerular filtration rate (mls/min/1.73m^2^)	72.4±20.1	78.5±19.0	71.8±20.1	<0.001	0.601***
Atrial fibrillation	42 (2.3%)	0 (0.0%)	42 (2.6%)	0.028*	4.374**
Heart failure	243 (13.5%)	0 (0.0%)	243 (14.9%)	<0.001	28.559**
HFrEF	133 (7.4%)	0 (0.0%)	133 (8.1%)		
HFmrEF	35 (1.9%)	0 (0.0%)	35 (2.1%)		
HFpEF	75 (4.2%)	0 (0.0%)	75 (4.6%)		
Stroke	97 (5.4%)	0 (0.0%)	97 (5.9%)	0.001	10.422**
Chronic kidney disease	37 (2.1%)	0 (0.0%)	37 (2.3%)	0.043*	3.840**
Coronary artery disease	33 (1.8%)	1 (0.6%)	32 (2.0%)	0.358*	1.541**
Calcium channel blockers	1239 (68.9%)	114 (68.7%)	1125 (68.9%)	0.954	0.003**
Diuretics	954 (53.0%)	5 (45.2%)	879 (53.8%)	0.033	4.523**
ACEIs	733 (40.7%)	57 (34.3%)	676 (41.4%)	0.078	3.110**
ARBs	707 (39.3%)	51 (30.7%)	656 (40.2%)	0.018	5.639**
Beta-blockers	629 (35.0%)	52 (31.3%)	577 (35.3%)	0.302	1.065**
Alpha-blockers	73 (4.1%)	4 (2.4%)	69 (4.2%)	0.259	1.276**
Centrally acting	67 (3.7%)	10 (6.0%)	57 (3.5%)	0.101	2.698**
Arterial vasodilators	8 (0.4%)	0 (0.0%)	8 (0.5%)	0.366	0.817**
Statin use	647 (36.0%)	47 (28.3%)	600 (36.7%)	0.031	4.649**

Table 2 shows the pattern of risk factors and comorbidities among all patients by the presence or absence of LVH. In patients ≥60 years, more patients had LVH, while fewer patients had LVH in those <60 years (p<0.001). More female patients had LVH while fewer males had LVH (p=0.003). For comorbidities, patients with LVH had significantly more AF, HF, stroke, CKD, and LVDD (p<0.05). Patients with LVH had higher SBP (p=0.027), but there was no significant difference between the two groups for DM, obesity, DBP, and CAD (p>0.05). 

**Table 2:** Pattern of risk factors and comorbidities among all hypertensive patients by echocardiographic LVH. BMI: Body mass index; DBP: Diastolic blood pressure; ECHO: Echocardiographic; LV: Left ventricular; LVH: Left ventricular hypertrophy; SBP: Systolic blood pressure, **Pearson Chi-Square, ***Independent t-test.

Variables	Total (n=1799)	ECHO LVH absent, n=946 (52.6%)	ECHO LVH present, n=853 (47.4%)	p-value	Test value
Age (years)				<0.001	13.981**
<60 years	1098 (61.0%)	616 (65.1%)	482 (56.5%)		
≥60 years	701 (39.0%)	330 (34.9%)	371 (43.5%)		
Sex				0.003	8.693**
Male	793 (44.1%)	448 (47.4%)	345 (40.4%)		
Female	1006 (55.9%)	498 (52.6%)	508 (59.6%)		
Diabetes mellitus	363 (20.2%)	177 (18.8%)	186 (21.9%)	0.099	2.719**
Obesity (BMI≥30kg/m^2^)	888 (49.5%)	462 (48.9%)	426 (50.1%)	0.619	0.248**
First visit SBP (mmHg)	147.0±22.6	145.8±20.7	148.2±24.5	0.027	11.762***
First visit DBP (mmHg)	89.0±14.1	88.8±12.8	89.2±15.4	0.531	22.822***
LV diastolic dysfunction	1108 (61.6%)	480 (50.8%)	628 (73.6%)	<0.001	98.419**
Atrial fibrillation	42 (2.3%)	12 (1.3%)	30 (3.5%)	0.002	9.923**
Heart failure	243 (13.5%)	29 (3.1%)	214 (25.1%)	<0.001	186.202**
Stroke	97 (5.4%)	37 (3.9%)	60 (7.0%)	0.003	8.575**
Chronic kidney disease	37 (2.1%)	11 (1.2%)	26 (3.0%)	0.005	7.914**
Coronary artery disease	33 (1.8%)	17 (1.8%)	16 (1.9%)	0.901	0.015**

Table 3 shows the multivariable logistic regression of risk factors and comorbidities associated with LVH in our study population. There was a significant positive association between LVH and female sex, LVDD, HF, and stroke (p<0.05). There was no association with age, AF, and CKD (p>0.05).

**Table 3:** Multivariable logistic regression of risk factors and comorbidities among all patients with left ventricular hypertrophy. CI: Confidence interval; AOR: Adjusted odds ratio; B: Beta regression coefficient. Model fit p= 0.034, Cox & Snell R Square of 0.141, Nagelkerke R Square of 0.189, Hosmer and Lemeshow Test of 0.423.

	Left ventricular hypertrophy		
Co-variates	AOR (95% CI)	p-value	B
Age (≥ 60 years)	1.04 (0.83-1.29)	0.751	0.025
Sex (Female)	1.47 (1.20-1.80)	<0.001	0.383
Diastolic dysfunction	2.01 (1.61-2.50)	<0.001	0.702
Heart failure	9.71 (6.20-15.23)	<0.001	2.274
Atrial fibrillation	0.58 (0.25-1.33)	0.199	-0.544
Stroke	1.59 (1.01-2.52)	0.045	0.469
Chronic kidney disease	1.28 (0.55-2.98)	0.573	0.245

Table 4 compares risk factors and comorbidities among HHD patients with LVDD. In patients ≥60 years, more patients had LVDD, while more patients had normal LV diastolic function in those <60 years (p<0.001). More patients with DM, LVH, HF, stroke, and CKD had LVDD (p<0.05) while more patients with concentric LV remodeling had normal LV diastolic function (p<0.001). SBP was higher among those with LVDD (p=0.008) but there was no association between LVDD and obesity, AF, DBP, CAD, and sex (p>0.05). 

**Table 4:** Pattern of risk factors and comorbidities among patients with hypertensive heart disease with LV diastolic dysfunction. BMI: Body mass index; DBP: Diastolic blood pressure; LVDD: Left ventricular diastolic dysfunction; SBP: Systolic blood pressure, **Pearson Chi-Square, ***Independent t-test.

Variables	Total (n=1799)	LVDD absent, n=690 (38.4%)	LVDD present, n=1109 (61.6%)	p-value	Test value
Age (years)				<0.001	187.891**
<60 years	1098 (61.0%)	559 (81.0%)	539 (48.6%)		
≥60 years	701 (39.0%)	131 (19.0%)	570 (51.4%)		
Sex				0.397	0.747**
Male	793 (44.1%)	313 (45.4%)	480 (43.3%)		
Female	1006 (55.9%)	377 (54.6%)	628 (56.7%)		
Diabetes mellitus	363 (20.2%)	85 (12.4%)	278 (25.2%)	<0.001	42.929**
Obesity (BMI≥30 kg/m^2^)	888 (49.5%)	340 (49.3%)	548 (49.6%)	0.905	0.010**
First visit SBP (mmHg)	147.0±22.6	145.2±20.1	148.1±24.0	0.008	16.603***
First visit DBP (mmHg)	89.0±14.1	89.2±12.4	88.9±15.1	0.714	20.670***
Concentric LV remodeling	687 (38.2%)	299 (43.3%)	388 (35.0%)	<0.001	12.278**
Left ventricular hypertrophy	853 (47.4%)	225 (32.6%)	628 (56.7%)	<0.001	98.419**
Atrial fibrillation	42 (2.3%)	11 (1.6%)	31 (2.8%)	0.100	2.700**
Heart failure	243 (13.5%)	22 (3.2%)	221 (19.9%)	<0.001	102.018**
Stroke	97 (5.4%)	17 (2.5%)	80 (7.2%)	<0.001	18.813**
Chronic kidney disease	37 (2.1%)	7 (1.0%)	30 (2.7%)	0.014	6.035**
Coronary artery disease	33 (1.8%)	12 (2.7%)	21 (1.9%)	0.810	0.056**

Table 5 shows the multivariable logistic regression of covariates for LVDD among those with HHD. There was a significant positive association between LVDD and age, DM, LVH, concentric LV remodeling, and stroke (p<0.05). There was no association with AF and CKD (p>0.05).

**Table 5:** Multivariable logistic regression of risk factors and comorbidities among patients with hypertensive heart disease with diastolic dysfunction. CI: Confidence interval; AOR: Adjusted odds ratio; B: Beta regression coefficient. Model fit p= < 0.001, Cox & Snell R Square of 0.198, Nagelkerke R Square of 0.269, Hosmer and Lemeshow Test of 0.203.

	Left ventricular diastolic dysfunction		
Co-variates	AOR (95% CI)	P-value	B
Age (≥ 60 years)	3.92 (3.09-4.96)	<0.001	1.373
Diabetes mellitus	1.83 (1.37-2.44)	<0.001	0.599
Left ventricular hypertrophy	3.51 (2.53-4.87)	<0.001	1.493
Concentric remodeling	2.18 (1.58-3.01)	<0.001	0.761
Atrial fibrillation	0.57 (0.22-1.46)	0.242	0.202
Stroke	1.87 (1.06-3.32)	0.032	0.663
Chronic kidney disease	1.27 (0.51-3.21)	0.607	0.604

## Discussion

Our study shows that 90.8% of our hypertensive cohort have HHD. Other key findings include a higher rate of concentric LVH compared to eccentric LVH. Additionally, individuals with LVH have a nine-fold increased risk of HF, a two-fold increase in LVDD, a 1.47-fold increase among female patients, and a 1.59-fold increased risk of stroke.

The prevalence of HHD in our study is very high and may be overstated because our study is hospital-based. However, it likely reflects the higher prevalence of HHD in Nigeria and other parts of SSA than reported by population-wide studies based on HTN without HHD. This situation poses serious implications, including increased morbidity and mortality from arrhythmias, sudden cardiac death, and HF, as well as heightened healthcare costs. In a global assessment of HHD burden by Lu et al. [2], the highest prevalence rates were in Asia, East Asia, and America, with lower rates in Africa. This relatively low prevalence in Africa may reflect the use of population-based studies of SBP without HHD and lower healthcare access impacting disease detection, thereby underestimating the burden of the disease. Characterizing HHD requires relatively expensive diagnostic tools such as the ECG and echocardiography, which are not widely available in many SSA communities. The use of population-based studies that do not emphasize the identification of cardiac damage seen in HHD therefore underestimates the burden of HHD. Furthermore, estimates of the prevalence of HHD from African studies using LV geometry [9-13] or hypertensive heart failure only [22], which excluded other phenotypes of HHD, will be low. These underestimations may lead to treatment delay and graver complications of HF, stroke, CAD, sudden death, and all-cause mortality [1]. Accurate and prompt identification of HHD is therefore essential for effective preventive and therapeutic strategies such as achieving optimal BP targets and the use of combination hypertensive therapy that prevents or regresses HHD changes. Affordable portable echocardiography or AI-assisted ECG interpretation by trained physicians could improve detection in low-resource settings. 

In our hypertensive cohort, the prevalence of echocardiographic LVH, concentric LV remodeling, and LVDD was 47.4%, 38.2%, and 61.6%, respectively. Out of those with LVH, 31.8% had concentric LVH and 15.6% had eccentric LVH. In a review of 30 echocardiographic studies, Cuspidi et al. found a prevalence of echocardiographic LVH ranging from 36% to 41% [8]. Our study recorded a slightly higher value, possibly due to over 80.3% of their patients being Caucasian. While there is consensus in the literature that there are no significant racial differences in LVM among Caucasian people and black people [23], Drazner et al. in a population-based study showed a higher prevalence of LVH in American black people than their Caucasian counterparts [24]. In hospital-based studies of hypertensives in Nigeria and Tanzania, Aje et al. and Chillo et al. reported similar prevalence of LVH as our study [10,25]. Our study’s prevalence of 61.6% of LVDD is also like the 62% reported by Adamu et al. [12]. However, Adebayo et al. in another hospital-based Nigerian study reported a lower LVH prevalence of 32.9% compared to our study [11]. We used a less conservative LVMI cut-off of 115 and 95 g/m^2^ in men and women, respectively, which is similar to direct necropsy and clinical trial measurements [15,23]. Findings from our study agree with the reported prevalence of hypertensive LVH based on BSA of >40% [1,8] and therefore suggest that our prevalence may be more accurate than studies using higher LVH cut-offs.

This study found a higher prevalence of concentric LVH compared to eccentric LVH, similar to other studies in black African populations and to a concentric hypertrophic response compared with whites [5,10,11,26]. Both concentric and eccentric response may transition directly to HF, and the severity of LVH further increases the risk of HF [5]. Therefore, efforts should be geared towards preventing and treating LVH, regardless of the phenotype.

In our study of hypertensive patients, we found that those with LVH had a nine-fold increase in HF, as well as a two-fold increase in LVDD, and a 1.59-fold increase in stroke compared to those without LVH. This aligns with the Framingham Heart Study which showed a three-fifteen-fold increase in CV events, with the greatest risk for HF and stroke in patients with ECG LVH [27]. Therefore, early diagnosis and treatment of HTN and LVH are important to reduce CV events. Our study also showed a higher risk of LVH among women compared to men. Previous studies on hypertensive women have shown increased LVMI and concentric hypertrophy [5]. Disparities in social, economic, and structural factors have been suggested to be responsible for these sex-based differences [28].

In our study, HHD patients with DM, concentric LV remodeling, stroke, LVH, and age over 60 had a higher risk of LVDD. This aligns with studies that have shown an association between LVDD and CV outcomes in general and hypertensive populations [1,29]. These associations may also reflect shared pathophysiological mechanisms such as myocardial and vascular stiffness, fibrosis and increased afterload. In hypertensive patients, LVDD is a precursor to atherosclerotic CV events and HF, and it is an independent predictor of mortality [1]. Therefore, additional CV risks are present for patients with DM, stroke, abnormal LV geometric patterns and those over 60 years old. Moreover, the independent association of DM with LVDD indicates metabolic involvement, suggesting that markers of insulin resistance, such as the triglyceride-glucose index, may offer additional insights. Our study shows that concentric LV remodeling is more frequent in those with normal diastolic function (p<0.001), suggesting a transition from remodeling to LVH. This association which has been reported previously reflect early-stage HHD [1,5,6]. Preventing the worsening of LVDD or reducing it is an important therapeutic target for CV event reduction. Reduction of SBP to <130mmHg has been shown to improve echocardiographic LVDD by increased e’ and decreased E/e’ over six months [8], but a similar improvement in echocardiographic LVDD was not seen over two years despite regression of LVH [30]. The use of atenolol-bendroflumethiazide combination may have resulted in the failure of improvement in LVDD. Further studies are needed to examine the long-term effects of BP reduction on LVDD.

Our study showed that hypertensives with concentric LV remodeling and LVH had a 2.18-fold and a 3.51-fold greater risk of LVDD respectively. In the HyperGEN study, de Simone showed that the odds of abnormal relaxation were 2.3-fold greater among hypertensives with LV concentric geometry [29], and other studies have also shown that LVDD increases the risk of major CV events in hypertensives [1]. Promotion of early identification of LVDD could reduce LVDD associated CV events.

Our study has some limitations to be taken into consideration when interpreting our findings. Although this is a single-center Nigerian study and not population-based, our patients reflect Nigeria's multiethnic diversity. However, the results may be more applicable to urban Nigeria. Our sample size is relatively large and the true burden of HHD is better reflected with the use of echocardiography as done in this study compared with using estimates of SBP as a surrogate for HHD. Advanced imaging techniques like CMR are more accurate than echocardiography for diagnosing HHD phenotypes. However, CMR is expensive and was not available for this study. In contrast, speckle tracking is more accessible and can detect very early signs of myocardial dysfunction, making it a valuable tool for future studies to provide additional insights. Our study calls for multi-center hospital-based and population-based studies on the burden and phenotypes of HHD as well as prospective studies on LVDD progression and interventions in SSA populations. 

## Conclusions

This hospital-based study showed a high prevalence of HHD in the Nigerian population, providing important data to bridge the gap filled by SBP as a surrogate maker for HHD. This translates to increased CV risk, specifically of HF and stroke in this population. The high prevalence is not surprising given the relatively low levels of treatment and control of HTN in Nigeria and SSA. Targeted strategies to reverse this trend may include stricter BP targets, greater access to affordable medications, combination hypertensive therapy, and lifestyle interventions. Further prospective multicenter, population-wide African studies on HHD using cost-effective portable ECG, conventional and speckle-tracking echocardiography are warranted to explore the progression of abnormal geometry and LVDD in our hypertensive populations. This has the potential of identifying early markers of HHD and may aid reduction of CV morbidity and mortality in a timely fashion.

Our study also shows the prognostic importance of LVH and LVDD in HHD, with LVH showing the most predictive risk for HF and stroke. Concerted efforts are therefore needed to ensure the prevention and regression of these HHD phenotypes through adequate BP control and early initiation of RAAS blockers. Furthermore, our study advocates for investments in cost-effective imaging tools necessary for early diagnosis of HHD to allow timely initiation of HHD regressing therapy. Finally, a call for policymakers and healthcare providers to take decisive action in prioritizing investments in healthcare infrastructure and improving healthcare practices related to HHD is imperative.
